# Biological rhythm disturbance in remitted bipolar patients

**DOI:** 10.1186/2194-7511-1-6

**Published:** 2013-06-13

**Authors:** Adriane R Rosa, Mercè Comes, Carla Torrent, Brisa Solè, Maria Reinares, Isabella Pachiarotti, Manel Salamero, Flávio Kapczinski, Francesc Colom, Eduard Vieta

**Affiliations:** Bipolar Disorders Program, Institute of Neurosciences, Hospital Clinic, University of Barcelona, IDIBAPS, CIBERSAM, Villarroel 170, Barcelona, Catalonia, 08036 Spain; Bipolar Disorders Program and INCT for Translational Medicine, Hospital de Clínicas de Porto Alegre, Universidade Federal do Rio Grande do Sul, Ramiro Barcelos 2350, Porto Alegre, Rio Grande do Sul 90035903 Brazil; Centro Universitário La Salle, Rua Victor Barreto 2350, Canoas, Rio Grande do Sul 92010-000 Brazil; Clinical Psychology Department, Institute of Neurosciences, Hospital Clinic, IDIBAPS, University of Barcelona, Villarroel 170, Barcelona, 08036 Spain

**Keywords:** Bipolar disorder, Circadian rhythms, Biological rhythms, Sleep alterations, Functioning

## Abstract

**Background:**

Biological rhythm disturbance is common in bipolar patients and seems to affect the course and prognosis of the illness negatively. The main aim of the current study was to assess biological rhythms in remitted bipolar patients. We also assessed whether there was an association between clinical variables or functioning and biological rhythms in remitted bipolar participants.

**Methods:**

The Biological Rhythms Interview of Assessment in Neuropsychiatry (BRIAN) was used to assess biological rhythm disturbance. It is an 18-item interviewer-administered instrument which allows us to investigate the main areas related to circadian rhythm disturbance (sleep/social, activities, and eating pattern) in bipolar disorder.

**Results and discussion:**

Bipolar patients (*n* = 107) experienced greater biological rhythm alterations than the control group (*n* = 100) (BRIAN total scores 35.36 ± 7.11 vs. 32.48 ± 6.10, *t* = 6.912, *p* = 0.002, Cohen's *d* = 0.43, *r* = 0.21). In particular, patients were more impaired than the control group with regard to sleep/social (14.67 ± 4.14 vs. 13.49 ± 2.91, *t* = 10.61, *p* = 0.018, Cohen's *d* = 0.33, *r* = 0.16) and activity (8.49 ± 2.51 vs. 7.07 ± 2.13, *t* = 3.90, *p* = 0.001, Cohen's *d* = 0.61, *r* = 0.29) domains. Furthermore, a significant correlation was found between biological rhythms with residual depressive symptoms (*r* = 0.459, *p* < 0.001) and functioning (*r* = 0.432, *p* < 0.001). These findings suggest a potential link between biological rhythms and the pathophysiology of bipolar disorder. It highlights the importance of novel instruments (e.g., BRIAN) which allow us to assess biological rhythm disturbance in psychiatry. Finally, specific psychosocial interventions focused on lifestyle regularity may be considered as a supplemental treatment of bipolar illness episodes.

**Electronic supplementary material:**

The online version of this article (doi:10.1186/2194-7511-1-6) contains supplementary material, which is available to authorized users.

## Background

Evidence has suggested that biological rhythm disturbance is etiologically involved in bipolar disorder (Jones 2001; Murray and Harvey 2010). Changes in the sleep-wake cycle, indeed, are part of the diagnostic criteria for bipolar disorder. For example, there is a decreased need for sleep during manic episodes, whereas insomnia and hypersomnia are presumably found in depression (American Psychiatric Association 1994). Other biological rhythms, such as the daily profiles of body temperature, cortisol, thyrotropin, prolactin, growth hormone, and melatonin, are also disrupted in bipolar disorder (Dallaspezia and Benedetti 2011).

The social zeitgeber theory suggests that irregular circadian rhythms lead to mood episodes in vulnerable individuals (Grandin et al. 2006). Consistent with this concept, biological rhythm alterations have been associated with mood symptoms and, consequently, with increased risk of relapses in bipolar disorder (Frank et al. 2006; Gruber et al. 2011). Furthermore, biological rhythm impairment has been associated with poor functioning and quality of life (Giglio et al. 2010). Therefore, treatments based on biological rhythm stability as well as control exposure to environmental stimuli (e.g., light) may have therapeutic effects on illness episodes (Dallaspezia and Benedetti 2011; Heiler et al. 2011).

In summary, circadian rhythms have a ‘triple action’ on bipolar patients as they potentially have an etiological/triggering role, they are usually mentioned by our patients as a very reliable early warning sign of relapse, and their modification - by means of, e.g., behavioral therapy, psychoeducation,interpersonal social rhythm therapy, or certain drugs - may have a therapeutic effect.

Despite all of the above, few studies have assessed biological rhythms in remitted bipolar patients (Brill et al. 2011; Harvey et al. 2005). In addition, most of them investigated sleep function exclusively, failing to take into account the other sensible areas involved in biological rhythms (Gruber et al. 2009; Sylvia et al. 2012). For instance, one study compared bipolar I disorder patients (*n*=19) with age- and gender-matched healthy controls, reporting that affected individuals presented higher sleep abnormalities in both objective (actigraphy) and subjective measures than healthy control participants (Millar et al. 2004). There is increasing evidence, however, that social rhythms, defined by attendance at work, engagement in social activities or recreation, and exercise, may affect circadian regularity as a greater variability of social activities has been consistently related to sleep disturbance (Carney et al. 2006; Morgan 2003). The Biological Rhythms Interview of Assessment in Neuropsychiatry (BRIAN) is a novel instrument which allows us to assess the main aspects involved in biological rhythm disturbance (e.g., sleep, social, activity, and eating patterns) in bipolar patients (see Additional file 1).

The current study was carried out to assess biological rhythms in remitted bipolar patients and also to investigate possible correlations between biological rhythms, clinical course of illness, and residual mood symptoms.

## Methods

### Subjects

All patients (*n* = 107) were enrolled from the Barcelona Bipolar Disorders Unit at the Hospital Clinic in Barcelona (Spain). The inclusion criteria were (a) age >18 years, (b) fulfilling DSM-IV-TR criteria for bipolar I or bipolar II disorder, and (c) meeting remission criteria defined as a score ≤8 on the 17-item Hamilton Depression Rating Scale (HAM-D) and a score ≤5 on the Young Mania Rating Scale (YMRS) score for at least 6 months prior to the assessment of circadian rhythms. One hundred control participants matched by age, gender, and education with no psychiatric disorders that manifested interest in participating in the study were included. Most of them were currently working; very few were householder, hospital staff, or student. Both patients and controls were recruited from the hospital catchment area to ensure a similar socioeconomic pattern between groups.

### Instruments

#### Biological rhythms interview of assessment in neuropsychiatry

The BRIAN was developed by the Bipolar Disorder Program at the Hospital de Clínicas de Porto Alegre, Brazil, taking into account the main difficulties associated with rhythm disturbance in psychiatric patients, especially patients with bipolar disorder. The initial version of the BRIAN included 56 which were studied in a pilot study consisting of 30 healthy controls and 30 bipolar patients. After preliminary analysis, the scale was discussed with some experts in this field, some changes were made, and some items were rejected. Then, the final version consists of 18 items divided into three main areas related to circadian rhythm disturbance in psychiatric patients, namely sleep/social rhythms, activities, and eating pattern. In particular, the BRIAN assesses the frequency of problems related to the maintenance of circadian rhythm regularity. For instance, ‘Do you have problems falling asleep at your usual time? How frequently?’ (Items are rated using a 4-point scale: (1) = no at all, (2) = seldom, (3) = sometimes, and (4) = often). The total BRIAN scores range, hence, from 1 to 72, where the higher scores suggest severe circadian rhythm disturbance. The validity and reliability of the Portuguese BRIAN version in bipolar disorder are described by Giglio et al. (2009). The current study included a validation of the Spanish version of the BRIAN following the back-translation method (Guillemin et al. 1993). Psychometric properties of the Spanish BRIAN version are shown in the present study.

The Pittsburgh Sleep Quality Index (PSQI) was also administered both to patients and controls as a standard measure for sleep disturbance assessment (Buysse et al. 1989). The Functioning Assessment Short Test (FAST) was used to assess the functional outcome of patients (Rosa et al. 2007, 2011). Manic and depressive symptoms were evaluated with the YMRS and the HAM-D, respectively. Clinical and demographic data were assessed using a standardized protocol. The pattern of medication prescribed was also recorded.

The study was approved by the Hospital Clinic of Barcelona Ethics Committee. After receiving a complete verbal description of the study, written informed consent was obtained from all participants.

### Statistical analysis

Statistical analysis was performed using SPSS for Windows - Version 18.0. Group comparisons (patients and controls) were made using Student's *t* test and *χ*^2^ test when appropriated. Pearson's correlation coefficient was performed to examine the possible relationship between BRIAN scores, clinical variables, and functioning. Psychometric properties of the Spanish BRIAN version including internal consistency, concurrent validity, and feasibility were also examined.

## Results

**Figure 1:**
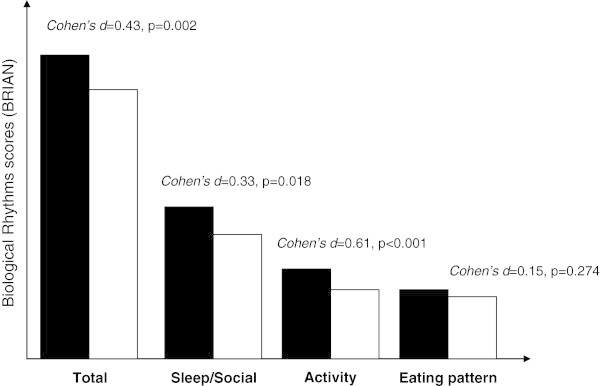
**Biological rhythm differences between patients and controls.**

Forty-four percent of the patients were female with an overall mean age of 43 ± 14.35 years, and 27.8% of them had high education level. Demographic and clinical data of the sample are presented in Tables 1 and 2, respectively. Bipolar patients experienced greater overall biological rhythm disturbance than the control group (BRIAN total scores 35.36 ± 7.11 vs. 32.48 ± 6.10, *t* = 6.912, *p* = 0.002, Cohen's *d* = 0.43, *r* = 0.21). Specifically, the patients were more impaired than the control group with regard to sleep/social (14.67 ± 4.14 vs. 13.49 ± 2.91, *t* = 10.61, *p* = 0.018, Cohen's *d* = 0.33, *r* = 0.16) and activity domains of the BRIAN (8.49 ± 2.51 vs. 7.07 ± 2.13, *t* = 3.90, *p* < 0.001, Cohen's *d* = 0.61, *r* = 0.29). No differences between groups were found regarding eating pattern (6.15 ± 2.33 vs. 5.81 ± 2.10, *t* = 1.74, *p* = 0.274, Cohen's *d* = 0.15, *r* = 0.076; see Figure 1).

**Table 1:** **Demographic characteristics of the sample**

	Patients (***N*** = 107)	Control (***N*** = 100)	***χ*** ^2^	***p*** value
	***n*** (%)	***n*** (%)		
Gender (female)	47 (43.9)	45 (45)	0.024	0.890
Education (highly qualified)	27 (27.8)	33 (36.7)	1.671	0.213
Employed	41 (62.1)	73 (75.3)	5.673	0.225
Marital status				
Married	24 (35.8)	54 (55.1)	5.984	0.05
Living together			12.732	0.005
Living alone	11 (16.7)	14 (14.3)		
Living with parents	21 (31.8)	11 (11.2)		
Living with own family	28 (42.4)	65 (66.3)		
Others	6 (9.1)	8 (8.2)		
Bipolar type I	66 (76.7)			
Seasonal pattern	33 (36.7)			
Psychotic symptoms	61 (64.2)			
Drug use disorder	83 (77.6)			
Lifetime events	53 (59.6)			
Family history of psychiatric disorders	66 (71)			
Family history of affective disorders	54 (59.3)			
Axis I comorbidity	39 (36.4)			
Axis II comorbidity	41 (38.3)			
Medications				
Mood stabilizers	84 (77.1%)			
Antipsychotics	68 (62.4%)			
Antidepressants	22 (20.2%)			
Benzodiazepines	25 (22.9%)

**Table 2:** **Clinical characteristics of the sample**

	Patients		Control		***t*** value	***p*** value
	Mean	SD	Mean	SD		
Age	42.88	14.35	43.1	13.68	−0.11	0.913
Age of onset	26.59	11.77				
Total episodes	10.97	12.37				
Manic episodes	2.54	3.98				
Depressive episodes	4.89	5.27				
Mixed episodes	0.48	1.15				
Hypomanic episodes	3.15	4.75				
Suicide attempts	1.74	0.44				
Number of hospitalization	1.54	1.58				
HAM-D	3.57	3.7				
YMRS	1.94	3.23				

SD, standard deviation.

With regard to the psychometric properties of the BRIAN, all items of the instrument were completed by the totality of the participants, indicating high feasibility. The internal consistency coefficient obtained was good with a Cronbach's alpha of 0.703. The total BRIAN scores were significantly correlated with the global PSQI (*r* = 0.349, *p* = 0.003), with the PSQI sleep quality (*r* = 0.357, *p* = 0.003), and with the PSQI daytime dysfunction (*r* = 0.422, *p* < 0.001) which suggest the concurrent validity of the instrument.

A significant correlation was found between overall biological rhythms and depressive symptoms (*r* = 0.459, *p* < 0.001) and functioning (*r* = 0.432, *p* < 0.001). Correlations between BRIAN subscores and clinical variables are shown in Table 3.

**Table 3:** **Correlations between biological rhythms, clinical course, mood symptoms, and functioning**

	BRIAN total	BRIAN sleep/social	BRIAN activity	BRIAN eating pattern
Age	0.106	0.118	0.157	−0.05
Age of onset	0.096	0.207*	0.026	−0.057
Total episodes	0.048	−0.024	0.246*	−0.054
Manic episodes	−0.043	−0.064	0.051	−0.011
Depressive episodes	0.095	−0.038	0.291**	0.042
Suicide attempts	−0.084	−0.015	−0.067	−0.066
Number of hospitalization	0.139	0.152	0.037	0.122
HAM-D	0.459**	0.401**	0.453**	0.158
YMRS	0.214	0.276**	−0.029	0.411
FAST	0.432**	0.254*	0.611*	0.177

*p<0.05; **p<0.01.

No differences were observed regarding the overall BRIAN scores in patients who were or were not on mood stabilizers, antipsychotics, antidepressants, and benzodiazepines treatment.

## Discussion

Our results show that patients experience greater biological rhythm disturbance than healthy controls. In particular, there are significant differences between groups in sleep/social and activity patterns. These findings are consistent with previous reports (Kapczinski et al. 2011; Plante and Winkelman 2008; Scott 2011) suggesting that biological rhythm dysregulation plays a critical role in the pathophysiology of bipolar disorder.

Biological rhythm abnormalities have also been reported in unipolar depression (Haynes et al. 2006), in individuals at high risk of bipolar spectrum disorders (Ankers and Jones 2009), in participants with bipolar spectrum disorders (Shen et al. 2008), and in rapid cycling bipolar patients (Ashman et al. 1999). In particular, Ashman et al. (1999) showed that patients with rapid cycling perform fewer activities including diet, work, and social activities compared to control participants. Using the Social Rhythm Metric, another study found that bipolar spectrum individuals (with cyclothymia or bipolar II disorder) experienced less regular daily activities than the control group. Additionally, social rhythm irregularity was associated with the onset of affective episodes as well as a worsening of long-term outcome (Shen et al. 2008). Significant differences were observed between individuals at high risk of bipolar spectrum disorders and healthy controls with regard to the relative amplitude of activity patterns and sleep parameters such as duration, fragmentation, and efficiency (Ankers and Jones 2009). Our results support previous findings showing that bipolar patients not only experienced sleep-wake alterations, but also were less regular in their personal relationships and work tasks. Disruptions in daily activities and social interactions could act as ‘social zeitgebers’ which may contribute to increased biological abnormalities (e.g., hormonal, metabolic). This may account in part for the association between lack of regularity and clinical symptomatology in bipolar disorder.

As demonstrated in previous works (Frank et al. 2005; Goldstein et al. 2008), we found a significant association between biological rhythm disturbance and depressive symptoms and poor functioning. In addition, patients with a greater variability in daily routines were more likely to have depressive episodes. However, it is unclear if rhythm abnormalities contribute to depressive symptoms or they are a consequence of mood symptoms. In this sense, results from a prospective study with a large sample of bipolar participants (*n* = 196) showed that lower sleep duration was associated with increased mania while greater sleep variability was associated with increased mania and depression severity as well as poor functioning (Gruber et al. 2009, 2011). Other researchers have also reported that sleep deficits predict depressive symptoms at a 6-month follow-up but was not predictive of manic episodes (Perlman et al. 2006). Furthermore, several reports demonstrated that interpersonal and social rhythm therapy focusing on regular routines of sleeping, waking, exercise, and social interaction seems to be effective in preventing relapses and improving functioning in bipolar patients (Frank et al. 2006, 2008). Taken together, all these findings highlight that those patients with biological rhythm abnormalities are more likely to experience concurrent depressive symptoms with chronic course and poorer prognosis. Biological rhythm stabilization may be an important target for interventions aiming to treat mood symptoms, prevent relapses, and improve functioning in bipolar disorder.

Our results should be interpreted cautiously for several reasons. First, all participants were in remission and followed in a specialized bipolar disorder care (Vieta et al. 2011). Additionally, many of the patients had previously received psychoeducation which may have increased their daily activity regularity. Second, although the BRIAN is an interviewer-administered instrument, it is also based on patients' self-report, which may have influenced the results. Further studies are needed to investigate the possible correlations between subjective (BRIAN) and objective measures (e.g., polysomnography). Third, this is a cross-sectional study which does not allow us to determine the direction of the relationship between circadian abnormalities and mood symptoms. Longitudinal studies are needed to clarify this issue. The use of more objective assessment tools (actigraphy) should be considered, although this technique is not free of potential biases, especially regarding the evaluation of sleep (Spruyt et al. 2011).

## Conclusions

This study showed that biological rhythm disturbance (e.g., sleep, social, and activity patterns) is common in bipolar patients, suggesting a crucial link between rhythm instability and bipolar disorder. These findings highlight the importance of a more comprehensive assessment of biological rhythms in psychiatric disorders, which can be achieved by administering the BRIAN scale. Finally, specific psychosocial interventions focused on lifestyle regularity should be considered as an add-on maintenance treatment for bipolar disorder.

## Electronic supplementary material

Additional file 1: **Biological Rhythm Interview of Assessment in Neuropsychiatry (BRIAN).** The file contains questions on the main aspects involved in biological rhythm disturbance. (PDF 17 KB)
