# Supplementary material for: Biological rhythm disturbance in remitted bipolar patients
Source: Int J Bipolar Disord. 2013 Jun 13;1:6. doi: 10.1186/2194-7511-1-6 (PMC4230817; doi:10.1186/2194-7511-1-6)
Supplement: Supplementary file 1 — Additional file 1: Biological Rhythm Interview of Assessment in Neuropsychiatry (BRIAN). The file contains questions on the main aspects involved in biological rhythm disturbance. (PDF 17 KB) [file 40345_2012_6_MOESM1_ESM.pdf]

BIOLOGICAL RYTHMS INTERVIEW OF ASSESSMENT IN NEUROPSYCHIATRY (BRIAN)

De los aspectos que se indican a continuación señale la opción que escribe mejor la conducta del paciente en los últimos 15 días.

SUEÑO

1. ¿En qué medida le cuesta dormirse a la hora habitual?
(1) nada
(2) poco
(3) bastante
(4) mucho

2. ¿En qué medida le cuesta despertarse a la hora habitual?
(1) nada
(2) poco
(3) bastante
(4) mucho

3. ¿En qué medida le cuesta levantarse de la cama después de haberse despertado?
(1) nada
(2) poco
(3) bastante
(4) mucho

4. ¿En qué medida le cuesta sentirse descansado con el número de horas qué duerme (esto haría referencia a la sensación subjetiva de estar descansado para el desempeño de las tareas diarias como conducir, razonar o trabajar)?
(1) nada
(2) poco
(3) bastante
(4) mucho

5. ¿En qué medida le cuesta "desconectar" en los momentos de descanso?
(1) nada
(2) poco
(3) bastante
(4) mucho

ACTIVIDADES

6. ¿En qué medida le cuesta llevar a cabo todas las actividades que hace en el trabajo?
(1) nada
(2) poco
(3) bastante
(4) mucho

7. ¿En qué medida le cuesta llevar a cabo sus actividades habituales (limpiar la casa, hacer la compra)?
(1) nada
(2) poco
(3) bastante
(4) mucho

8. ¿En qué medida le cuesta mantener su ritmo de actividad física (por ejemplo: tomar un autobús/metro, o practicar deporte – si esto forma parte de su rutina)?
(1) nada
(2) poco
(3) bastante
(4) mucho

9. ¿En qué medida le cuesta cumplir el horario habitual de sus tareas?
(1) nada
(2) poco
(3) bastante
(4) mucho

10. ¿En que medida le cuesta mantener su nivel de deseo/actividad sexual?
(1) nada
(2) poco
(3) bastante
(4) mucho

SOCIAL

11. ¿En qué medida le cuesta relacionarse y comunicarse con las personas de su entorno?

(1) nada
(2) poco
(3) bastante
(4) mucho

12. ¿En qué medida le cuesta no abusar del uso de aparatos electrónicos como la TV, internet, etc. (sin que ello afecte a su contacto con las personas de su entorno o le quite demasiado tiempo de otras actividades)?
(1) nada
(2) poco
(3) bastante
(4) mucho

13. ¿En qué medida le cuesta ajustar sus rutinas y patrón de sueño al de las personas con quienes convive (familiares, vecinos, amigos)?
(1) nada
(2) poco
(3) bastante
(4) mucho

14. ¿En qué medida le cuesta disponer de tiempo y atención para las personas con quienes convive (familiares, vecinos, amigos)?
(1) nada
(2) poco
(3) bastante
(4) mucho

ALIMENTACIÓN

15. ¿En qué medida le cuesta mantener el horario de sus comidas (desayuno, comida y cena)?
(1) nada
(2) poco
(3) bastante
(4) mucho

16. ¿En qué medida le cuesta mantener su patrón de alimentación habitual en lo referente a no saltarse comidas?
(1) nada
(2) poco
(3) bastante
(4) mucho

17. ¿En qué medida le cuesta mantener su patrón de alimentación habitual en lo referente a la cantidad de alimento ingerido?
(1) nada
(2) poco
(3) bastante
(4) mucho

18. ¿En qué medida le cuesta no consumir en exceso sustancias estimulantes (como café o coca-cola), o chocolates/dulces?
(1) nada
(2) poco
(3) bastante
(4) mucho

RITMO PREDOMINANTE (vespertino o matutino)
Esta parte de la escala es opcional y hace referencia a sus hábitos. Considere aquí los últimos 12 meses.

19. ¿Tiene usted tendencia a estar más activado por la noche (trabajo, relaciones interpersonales)?
(1) nunca
(2) raramente
(3) casi siempre
(4) siempre

20 ¿Tiene usted la sensación de estar más productivo por la mañana?
(1) nunca
(2) raramente
(3) casi siempre
(4) siempre

21. ¿Tiene usted la sensación de cambiar el día por la noche?
(1) nunca
(2) raramente
(3) casi siempre
(4) siempre

BIOLOGICAL RYTHM INTERVIEW OF ASSESSMENT IN NEUROPSYCHIATRY – BRIAN

From the options below, check the one that better describes the patient's behavior *in the last 15 days*.

SLEEP

(1) Do you have problems falling asleep at your usual time? How frequently?

1. Not at all 2. Seldom 3. Sometimes 4. Often

(2) Do you have problems waking up at your usual time? How frequently?

1. Not at all 2. Seldom 3. Sometimes 4. Often

(3) Do you have problems getting out of bed after you wake up? How frequently?

1. Not at all 2. Seldom 3. Sometimes 4. Often

(4) Do you feel rested with the amount of sleep you usually get (including subjective perception of being rested and actual performance in daily activities, such as driving, working)? How frequently?

1. Not at all 2. Seldom 3. Sometimes 4. Often

(5) Do you fell you have difficulty switching off at the time of resting? How frequently?

1. Not at all 2. Seldom 3. Sometimes 4. Often

ACTIVITY

(6) Do you have difficulties in completing activities at your work? How frequently?

1. Not at all 2. Seldom 3. Sometimes 4. Often

(7) Do you have difficulties in completing your household activities? How frequently?

1. Not at all 2. Seldom 3. Sometimes 4. Often

(8) Do you have difficulties in keeping your usual rhythm of physical activity (e.g. taking a bus, metro or practicing sports)? How frequently?

1. Not at all 2. Seldom 3. Sometimes 4. Often

(9) Do you have difficulties in performing your daily activities in the time scheduled? How frequently?

1. Not at all 2. Seldom 3. Sometimes 4. Often

(10) Do you have difficulties in keeping your usual level of libido / sexual activity? How frequently?

1. Not at all 2. Seldom 3. Sometimes 4. Often

SOCIAL

(11) Do you have difficulties in communicating and having interpersonal relationships with significant others? How frequently?

1. Not at all 2. Seldom 3. Sometimes 4. Often

(12) Do you overly use electronic devices to the point that it impairs your interpersonal relationships (such as the television or the internet)? How frequently?

1. Not at all 2. Seldom 3. Sometimes 4. Often

(13) Do you have difficulties in synchronizing daily routines and sleep patterns with significant others (family, friends, spouse)? How frequently?

1. Not at all 2. Seldom 3. Sometimes 4. Often

(14) Do you have difficulties in giving attention to significant others (family, friends, spouse)? How frequently?

1. Not at all 2. Seldom 3. Sometimes 4. Often

EATING PATTERN

(15) Do you have difficulties in keeping the scheduled times for meals? How frequently?

1. Not at all 2. Seldom 3. Sometimes 4. Often

(16) Do you skip meals? How frequently?

1. Not at all 2. Seldom 3. Sometimes 4. Often

(17) Do you have difficulties in eating a regular amount during meals? How frequently?

1. Not at all 2. Seldom 3. Sometimes 4. Often

(18) Do you have difficulties in using stimulants with moderation? (such as coffee, coke and chocolate). How frequently?

1. Not at all 2. Seldom 3. Sometimes 4. Often

PREDOMINANT RHYTHM (CHRONOTYPE)

Consider *the last 12 months* for the following questions.

(19) Tends to be more energized for work and interpersonal relationships at night.

1. Never 2. Seldom 3. Often 4. Always

(20) Feels more productive in the morning.

1. Never 2. Seldom 3. Often 4. Always

(21) Do you have your day/night cycle reversed?

1. Never 2. Seldom 3. Often 4. Always
